# Linking brain activation to clinical outcomes: an fNIRS study in cochlear implant users and normal hearing individuals

**DOI:** 10.1117/1.NPh.13.S1.S13004

**Published:** 2025-09-11

**Authors:** András Bálint, Wilhelm Wimmer, Christian Rummel, Marco Caversaccio, Stefan Weder

**Affiliations:** a University of Bern, ARTORG Center for Biomedical Engineering Research, Hearing Research Laboratory, Bern, Switzerland; b Bern University Hospital, University of Bern, Inselspital, Department of ENT – Head and Neck Surgery, Bern, Switzerland; c Technical University of Munich, Klinikum rechts der Isar, Department of Otorhinolaryngology, Munich, Germany; d Bern University Hospital, University of Bern, University Institute of Diagnostic and Interventional Neuroradiology, Inselspital, Support Center for Advanced Neuroimaging (SCAN), Bern, Switzerland; e Technische Hochschule Deggendorf, European Campus Rottal-Inn, Pfarrkirchen, Germany

**Keywords:** cochlear implant, speech understanding, functional near-infrared spectroscopy, brain imaging, brain plasticity

## Abstract

**Significance:**

Cochlear implants (CIs) are a proven intervention for severe hearing loss; however, outcomes vary widely among CI recipients. Emerging evidence suggests that cortical adaptation to the electric hearing provided by CIs play a crucial role.

**Aim:**

We investigate cortical brain activation differences in CI users, comparing individuals with good speech understanding (good performers, GP) to those with poor outcomes (poor performers, PP) alongside a control group with normal hearing (NH).

**Approach:**

We recruited 46 CI users and 26 NH participants to perform a clinically adapted audiovisual speech comprehension task while we measured their brain activity using functional near-infrared spectroscopy (fNIRS). We corroborated our findings with objective and behavioral data.

**Results:**

Our findings showed distinct brain activation patterns associated with speech understanding. GP showed comparable brain activation patterns to NH in audio-only conditions, indicative of successful hearing rehabilitation. Further, both GP and PP participants showed an adaptive mechanism during visual speech processing. However, compared with GP, PP relied heavily on visual cues and showed altered neural resource allocation during audio-only conditions, potentially limiting their overall rehabilitation success.

**Conclusions:**

fNIRS revealed significant differences in brain activation between GP and PP, highlighting the role of cortical factors in CI rehabilitation. Understanding these neural mechanisms has the potential to lead to better patient counseling, optimized postoperative management, and personalized therapeutic strategies to improve outcomes for CI users.

## 1 Introduction

According to the World Health Organization, disabling hearing loss is a major communication and health problem currently affecting more than 430 million people worldwide, a prevalence that is expected to increase to more than 700 million by 2050.^1^ Among those affected, ∼50 million individuals suffer from a severe-to-profound form of hearing loss, a condition that cannot be adequately addressed with conventional hearing aids.^2^ Cochlear implants (CIs) are medical devices designed to restore hearing in individuals with severe-to-profound sensorineural hearing loss. Unlike hearing aids, which amplify sound, CIs bypass damaged portions of the inner ear and directly stimulate the auditory nerve by electric stimulation.^3^ Multicenter studies have shown that most CI recipients experience significant benefits. However, variability in postoperative speech understanding remains, with approximately one-third of patients falling short of preoperative expectations.^4^^–^^6^

The variability in CI outcomes has been extensively studied, with research identifying a range of contributing factors, such as the percentage of active electrodes at CI activation, the angle of electrode array insertion, and the cause of hearing loss.^5^^,^^7^^,^^8^ In addition, the progression of hearing loss contributes through factors such as the use of hearing aids during periods of moderate hearing loss, age at which severe-to-profound deafness began, the age at implantation, duration of severe hearing loss, pre-implant speech perception scores, and the pure tone average (PTA) hearing threshold of the better ear.^4^^,^^5^^,^^7^^,^^9^^–^^11^ Despite these findings, the factors identified so far attributed merely 10% to 31% of the total variance,^9^ underscoring the complexity of the underlying processes. This suggests that additional, yet unidentified mechanisms likely contribute to the observed variability.

Emerging evidence points to cortical processes as a key factor in this unexplained variance. Thereby, auditory deprivation appears to be a critical mechanism linking the progression of hearing loss to CI outcomes.^4^^,^^5^^,^^12^^,^^13^ The absence or reduction of auditory input leads to compensatory changes in the brain through cross-modal reorganization of brain networks driven by neural plasticity. Other sensory systems, such as the visual and somatosensory systems, may try to compensate for the missing auditory input.^14^^–^^17^ Following surgery, the auditory system is stimulated with electrical impulses from the surgically implanted electrode. Patients must then learn to interpret these electrical signals, a process that typically spans several months. During this adaptation phase, the brain adjusts to the new conditions.^12^^,^^18^^–^^20^ However, if nonauditory functions have taken over the auditory cortical regions, the brain’s limited ability to adapt may hinder the full effectiveness of sensory restoration.^18^

Researching neural adaptation is inherently complex and is further compounded by the diversity of study methodologies.^21^^–^^29^ Moreover, there is often a gap between standard clinical testing procedures, and the neuroimaging experiments conducted in research settings, making direct comparisons challenging. For this reason, it is crucial that stimulation protocols for neural activation studies are conducted under standardized, evaluated, and clinically relevant conditions, ensuring comparability with clinical assessments. In addition, behavioral parameters should be integrated into the data interpretation to enhance the reliability of the findings.

In this study, we adapted the well-established and validated German Matrix Sentence Test (OLSA) into a neuroimaging speech paradigm.^30^ This approach offers multiple advantages: (i) standardized testing conditions, (ii) a multimodal assessment of speech perception (audio, visual, and audiovisual speech understanding), (iii) clinical applicability, as the OLSA test is routinely used for CI evaluation and follow-up, and (iv) direct comparability between clinical findings and brain activation patterns. Using this multimodal OLSA test, we conducted brain activation measurements employing functional near-infrared spectroscopy (fNIRS). Three cohorts participated: normal hearing individuals (NH, controls), CI users with good speech understanding (GP, good performers), and CI users with poor speech understanding (PP, poor performers). Neuroimaging data were complemented by objective measures (e.g., hearing tests) and behavioral assessments (e.g., listening effort, in-task comprehension questions).

For the GP group, we hypothesized that successful hearing rehabilitation would be reflected by brain activation patterns similar to those of the NH group in temporal regions during listening tasks. In the audiovisual condition, we expected that GP participants might use visual cues to complement speech understanding, integrating these with auditory information effectively.^31^^–^^33^ For the PP group, we anticipated difficulties in listening conditions, potentially reflected by altered activation in temporal regions compared to NH.^21^ We further expected a stronger reliance on complementary visual cues. In an audiovisual condition, this reliance may lead to a different balance between auditory and visual processing compared to the GP group.

The aim of our study was to compare brain activation patterns between good and poor performing CI users and normal hearing individuals. By doing so, we sought to further improve insights into the variability of speech understanding in CI users. Ultimately, identifying specific biomarkers that reflect performance differences may help advance our understanding of the neural mechanisms essential for effective prosthetic hearing rehabilitation.

## 2 Materials and Methods

This study adhered to the ethical standards outlined in the Declaration of Helsinki. The study protocol (number 2020-02978) received approval from the local institutional review board (KEK-Bern). All participants provided informed consent prior to their involvement in the study. Forty-six individuals with CIs and 26 NH controls were recruited. CI users had PTA worse than 80 dB hearing level (HL) in both ears and were required to have been implanted for at least 6 months to ensure that the brain activation study did not interfere with the early rehabilitation phase. All participants were native German speakers with normal or corrected-to-normal vision and had no history of psychiatric, or neurological conditions, or brain injuries.

### 2.1 Audiological Assessment and Questionnaires

All audiological assessments took place in a sound-treated acoustic chamber. We performed a pure-tone audiogram (using a GSI 61, Grason-Stadler audiometer and headphone) and reported the PTA hearing thresholds (in dB HL).

To assess speech perception in quiet, we performed the Freiburg monosyllabic word test.^34^ In this test, the participants listened to monosyllabic words presented from a loudspeaker at 65 dB SPL, and were asked to repeat each word. The word recognition score (WRS) was calculated as the percentage of correctly repeated words.

To assess speech perception in noise, we performed the adaptive OLSA test.^35^^–^^37^ Participants listened to sentences presented from a loudspeaker at 65 dB SPL in the presence of background noise (OLnoise^38^), with the loudness adjusted based on the participants’ responses. The sentences followed a fixed five-word structure (name, verb, numeral, adjective, noun), drawn from a large inventory. This test determined the speech reception threshold (SRT), which is the minimum signal-to-noise ratio (SNR) where a person can correctly repeat at least 50% of the words.

We used the SRT to classify the participants. According to the visual analysis of the SRT distribution, a gap at ∼10  dB SNR was used to separate groups with clear differences in speech understanding [see Fig. 2(a)]. In the PP group, SRTs frequently exceeded 30 dB SNR and caused distortion in the loudspeaker output. Therefore, 30 dB SNR was set as the maximum level.

In addition to the audiological assessments, participants were asked to complete the following questionnaires: the Edinburgh Handedness Inventory,^40^ the Adaptive Categorical Listening Effort Scaling (ACALES) to assess listening effort,^39^ and an evaluation of lipreading abilities.^41^

### 2.2 Measurements of Brain Activity

We used fNIRS to measure cortical brain activation patterns. This technique measures changes in the concentration of oxygenated hemoglobin (HbO) and deoxygenated hemoglobin (HbR), reflecting local neural activity. The main advantage of fNIRS is its suitability for CI patients, even with active implants.^42^^–^^44^ We used the FOIRE-3000 continuous-wave fNIRS system (Shimadzu, Kyoto, Japan), which has 16 light sources and 16 light detectors (optodes). Thirty-five measurement channels were used for analysis, along with three short channels above the left and right temporal and occipital regions. The sampling rate was 14 Hz.

**Fig. 1:**
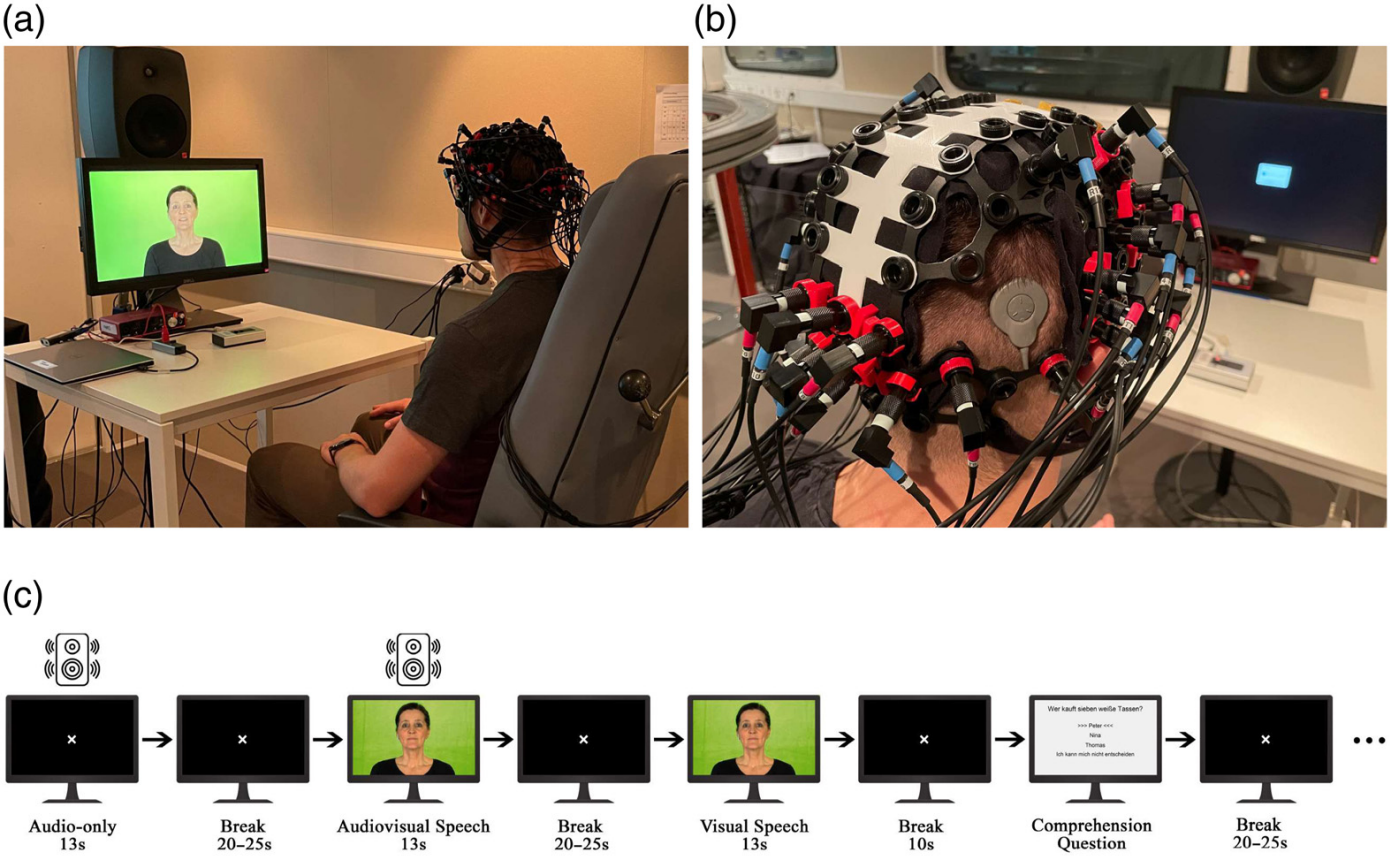
Experiment protocol. (a) Participants performed the audiovisual speech comprehension task in a sound-treated booth. (b) Placement of the optodes with the cochlear implant, using a custom optode holder cap. (c) The sentences were presented in the following conditions: audio-only, audiovisual, and visual speech. The sentences were followed by an additional comprehension question, presented in a counterbalanced block design.

Participants were seated in a darkened, sound-treated booth. Stimuli were presented using a monitor and a loudspeaker in a free-field setup, calibrated to 65 dB sound pressure level (SPL) [Fig. 1(a)]. The procedure was thoroughly explained to the participants, followed by a familiarization session. Once comfortable, the optode holder cap was fitted and adjusted to the participant’s head circumference. Optodes interfering with the position of the CI transmitter were removed [Fig. 1(b)]. NH participants listened with their natural hearing, whereas CI participants used the audio processor on their better-hearing side, based on hearing tests or their preference.

In the initial step, resting-state brain activity was recorded. Participants were instructed to close their eyes and relax for 5 min without falling asleep. Subsequently, participants listened to OLSA sentences (presented at 65 dB SPL without background noise) and answered comprehension questions presented in a counterbalanced block design [Fig. 1(c)]. Each sentence was repeated 3 times, resulting in a 13-s stimulus. Sentences were presented in three conditions: (1) audio-only speech (no video, fixation cross only), (2) audiovisual speech (audio and video), and (3) visual speech (video-only, i.e., lipreading). Each block consisted of one of each condition, followed by a comprehension question.

Each stimulus was followed by a 20 to 25 s nonstimulus interval, reduced to 10 s for comprehension questions. In total, 10 counterbalanced blocks were presented, resulting in 10 repetitions per condition and two comprehension questions per condition. Breaks were provided halfway through and at the end of the session to allow participants to relax and provide feedback on their listening effort.

### 2.3 Data Analysis

#### 2.3.1 Regions of interest

We defined regions of interest (ROIs) based on the neurocognitive model of auditory sentence processing by Love et al.^45^ Given the limited spatial resolution of fNIRS, we selected three ROIs: temporal, precentral, and occipital ROI, which are detailed in Table 1.

**Table 1:** Definition of regions of interest (ROIs).

Name of ROI	Brain regions	Function
Temporal	Middle temporal gyrus	Phonological processing of heard speech, lexical, and non-lexical lipreading^45^^,^^46^
	Superior temporal gyrus	
Precentral	Opercular and triangular part of the inferior frontal gyrus and precentral gyrus	Lexical lipreading, auditory-to-motor mapping^46^^,^^47^
Occipital	Superior occipital gyrus	Visually perceived information^48^^,^^49^
	Inferior occipital gyrus	
	Middle occipital gyrus	

To determine the individual optode positions, a 3D scan (Structure Sensor Pro, Occipital Inc., Boulder, United States) was conducted at the end of the fNIRS experiment.^50^^,^^51^ The optode positions were then registered onto the Automated Anatomical Labeling (AAL) Atlas available in the SPM toolbox.^52^^–^^54^

**Fig. 3:**
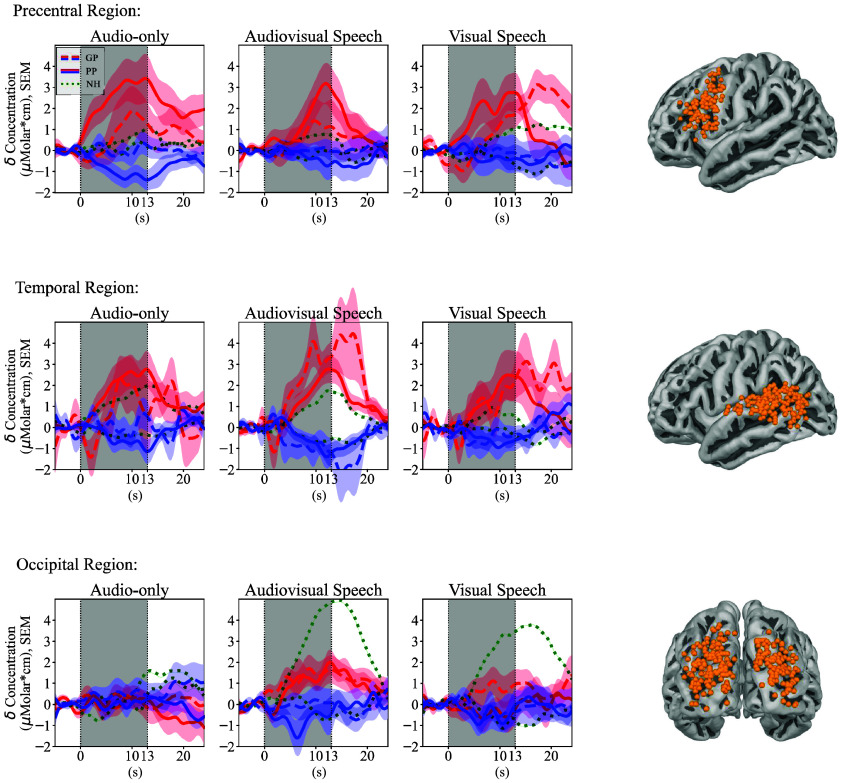
Grand average evoked responses in each region of interest (ROI) for each condition. The red signal represents the concentration change in oxygenated hemoglobin (HbO) and the blue signal in deoxygenated hemoglobin (HbR). Error bars represent the standard error of the mean (SEM). The dashed signals are measured in the good performing (GP), the solid signals in the poor performing (PP), and the green dotted signals in the normal hearing (NH) cohort, who were used as a control in the study. On the right side, we show all registered channel positions that were included in the corresponding ROI, indicating the brain regions contributing to the grand average patterns.

A measurement channel was included for further analysis if its position was registered to a brain region that was part of one of the predefined ROIs. The final set of channels corresponding to each ROI is visualized in Fig. 3. This method ensured that the analysis included only those channels that measured data from anatomically relevant brain regions.

#### 2.3.2 Data pre-processing

The fNIRS data preprocessing was performed using the HOMER2, NIRS Brain AnalyzIR, and MNE-NIRS toolboxes.^55^^–^^57^ First, we assessed the signal quality by analyzing the cardiac component of the HbO signal. The average peak of the Gaussian fit to the HbO frequency spectrum^58^^,^^59^ is reported in Appendix Table 5. Next, we conducted short-separation regression on the raw absorbance data using the nearest short channel.^58^^,^^60^^,^^61^ Motion artifacts were removed using the wavelet filter.^56^ The signal was then bandpass filtered in the range of 0.01 to 0.3 Hz and converted to changes in HbO and HbR concentrations based on the specifications of the fNIRS machine (resulting unit: μMolar*cm).^57^^,^^62^ Individual epochs from 0 to 24 s following the onset of stimulation were extracted and baseline corrected (−5 to 0 s). For the precentral and temporal ROIs, in CI participants, channels contralateral to the implant were analyzed, where the CI transmitter did not limit the optode positioning. In NH participants, activity was averaged across both hemispheres. In the occipital ROIs, all channels were used.

#### 2.3.3 Statistical analysis

We characterized the brain activation signals using general linear modeling (GLM).^57^^,^^63^ The SPM-based canonical hemodynamic response function was fitted to the HbO epochs, as this chromophore has greater sensitivity to task-evoked changes.^64^^–^^66^ Following this step, we averaged the GLM coefficients (Theta) from the individual channels into ROIs. The averaging process was weighted by the inverse of the standard error from the GLM fit.^56^

For the group-level analysis of the fNIRS data, we followed three main steps:

(i) We examined the overall morphology of the hemodynamic responses by calculating the grand averages for each condition and ROI, focusing on changes in HbO and HbR concentrations. This served as a quality control measure, as a typical hemodynamic response is characterized by an increase in HbO and a corresponding decrease in HbR within the activated brain region, with a delay following the onset of stimulation.^67^
(ii) We analyzed differences between the CI groups (GP, PP) and the control group (NH) using linear mixed-effects (LME) models. The model included fixed effects for group and condition, as well as their interaction, with the NH group as the intercept. The participants were defined as a random effect. We defined one LME model for each ROI.^26^^,^^56^
(iii) We compared activation patterns within the CI groups (GP versus PP). In the LME analysis, we used the GP group as the intercept while keeping the same model parameters as previously.

To control for possible differences between the groups, we included additional covariates in the LME model (age, sex, and etiology).^68^ Further, to characterize the effect of handedness, we repeated the analysis in the Appendix without purely left-handed participants.

In addition to fNIRS measurements, we integrated additional variables to support a more comprehensive understanding of the brain activation data:

• Objective measures: these included hearing test scores.^34^^,^^37^
• Behavioral data: we analyzed listening effort ratings,^39^ task performance (accuracy and reaction time), and subjective measures such as self-reported lipreading experience.^41^

To statistically compare our non-imaging data, we used Pearson’s correlation and T-statistics.

## 3 Results

**Fig. 2:**
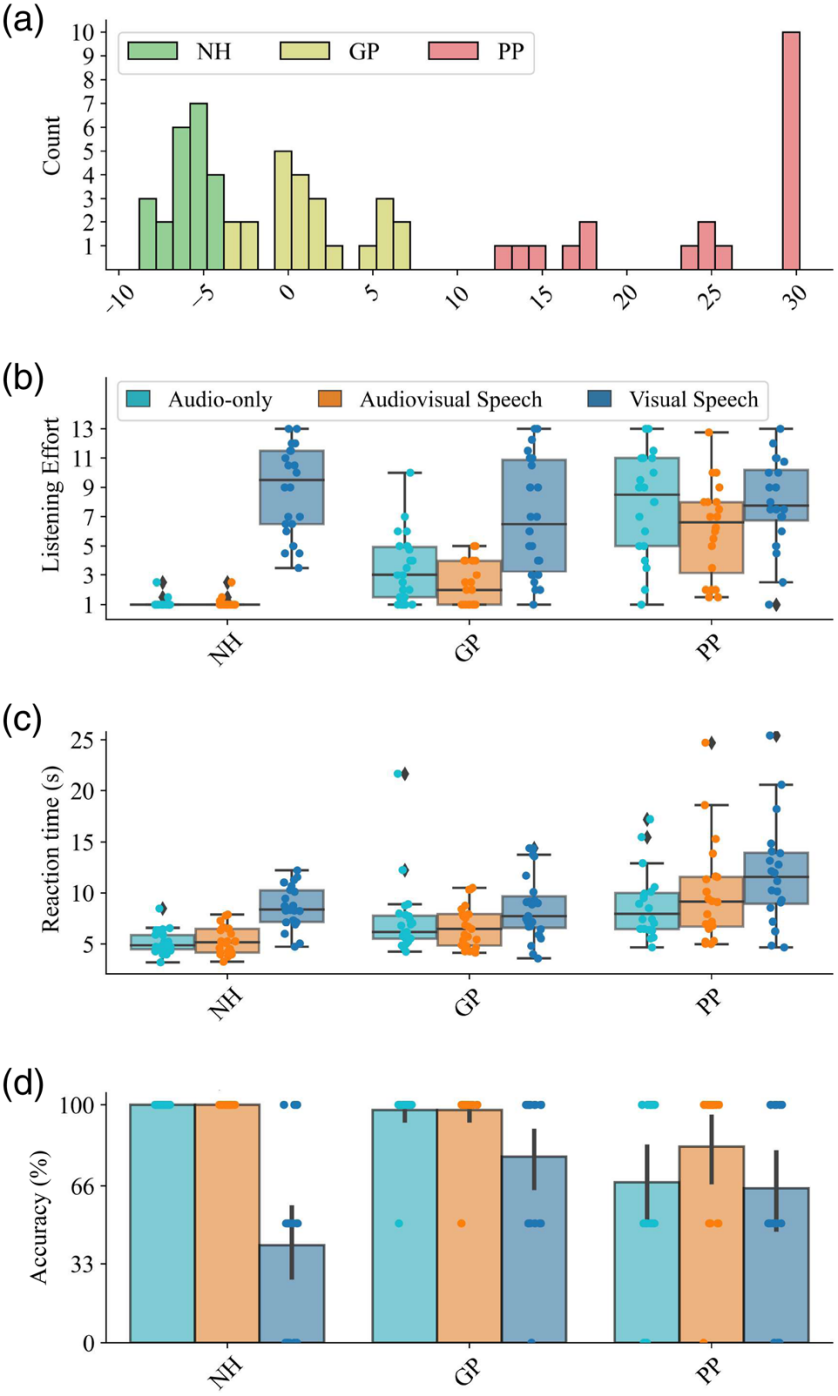
Summary of objective and behavioral variables. Panel (a) is the participant distribution by speech reception threshold (SRT). Panel (b) is the reported listening effort based on the Adaptive Categorical Listening Effort Scaling (ACALES).^39^ Panel (c) is the reaction time to the comprehension questions, and panel (d) is the task accuracy described by the percentage of correct answers to the comprehension questions. The box plot shows the median, quartiles, data range, and outliers. The corresponding t-statistics are summarized in Table 8 in the Appendix.

Seventy-two participants were included in the study. Twenty-five CI users were classified into the GP group and 21 into the PP group. The two groups were clearly separated by a margin in SRT as depicted in Fig. 2. In addition, 26 NH subjects were recruited as controls.

Seven participants were excluded from the final analysis: four due to poor signal quality (3 NH, 1 PP) and three due to inability to follow the task (1 NH, 2 GP). Participant characteristics are summarized at the group level in Table 2, in Fig. 2, and at the individual level in Table 5 in the Appendix.

**Table 2:** Overview of participant demographics.

	NH	GP	PP
**Participants**			
Total no.	26	25	21
Excluded:	4	2	1
Sex (male to female)	17:9	8:17	10:11
Age^a^	31.1 (5.86), 24−47	53.0 (18.77), 22−78	54.4 (16.78), 19−72
**Medical history**			
PTA^a^	6.4 dB HL (3.8), (−1.2) to 14.4	≥80 dB HL	≥80 dB HL
WRS^a^	100% for everyone	88.2% (10.4), 60−100	34.8% (21.76), 0−85
SRT^a^	(−6.1) (1.21), (−9) to (−4)	1.3 (3.0), (3)−7	24.8 (6.5), 13−30
CI side	N/A	L: 10, R: 15	L: 11, R: 10
Etiology	N/A	Pre: 6, Post: 19	Pre: 12, Post: 9
**Questionnaires**			
Handedness	L: 1, R: 23, A: 2	L: 2, R: 21, A: 2	L: 1, R: 17, A: 3
Lipreading^a^	0.8 (1.02), 0–3	4.9 (2.82), 0–10	7.4 (2.27), 2–10

PTA: pure-tone average (PTA) hearing thresholds (in decibel hearing level (dB HL)) at 500, 1000, 2000, and 4000 Hz.

WRS: results of the Freiburg monosyllabic word test at 65 dB sound pressure level (SPL).

SRT: speech reception threshold (SRT) in 65 dB SPL background noise using the adaptive OLSA test.

CI side: the side used during the fNIRS measurement.

Etiology: onset of deafness in relation to language development.

Handedness: L, left-handed; R, right-handed; A, ambidextrous.

Lipreading: self-reported lipreading experience on a scale from 0 to 10.

a Notation: mean (SD), range

### 3.1 Behavioral Data

The behavioral measures revealed distinct patterns in participants’ task performance and listening effort across different conditions (summarized in Fig. 2 and Table 8 in the Appendix).

The NH participants had no listening effort in the audio-only and audiovisual conditions. This was also evident in the short reaction times and correct answers to the comprehension questions. In the visual speech condition, however, the listening effort was high, the reaction time increased and the task accuracy was at chance level (1/3). Inexperience with lipreading was also reflected in the questionnaire (on average 0.8 rating out of 10, where 0 meant no experience in lipreading and 10 meant extensive skills in lipreading).

The GPs scored similarly to the NH group; however, there were also notable differences. Listening to audio-only and audiovisual speech was associated with significantly higher listening effort. Reaction times were comparable in all conditions; however, task accuracy was significantly higher for visual speech. The lipreading experience was higher than NH but less than PP (4.9 out of 10).

PP participants exhibited significantly higher listening effort in both audio-only and audiovisual conditions than the other two groups. This was paired with significantly longer reaction times than NH in both conditions. Task accuracy was generally lower than that of the other groups in the listening tasks, whereas for visual speech, it fell between the NH and GP participants. Despite their challenges, PPs rated their lipreading experience high, with an average score of 7.4.

**Fig 4:**
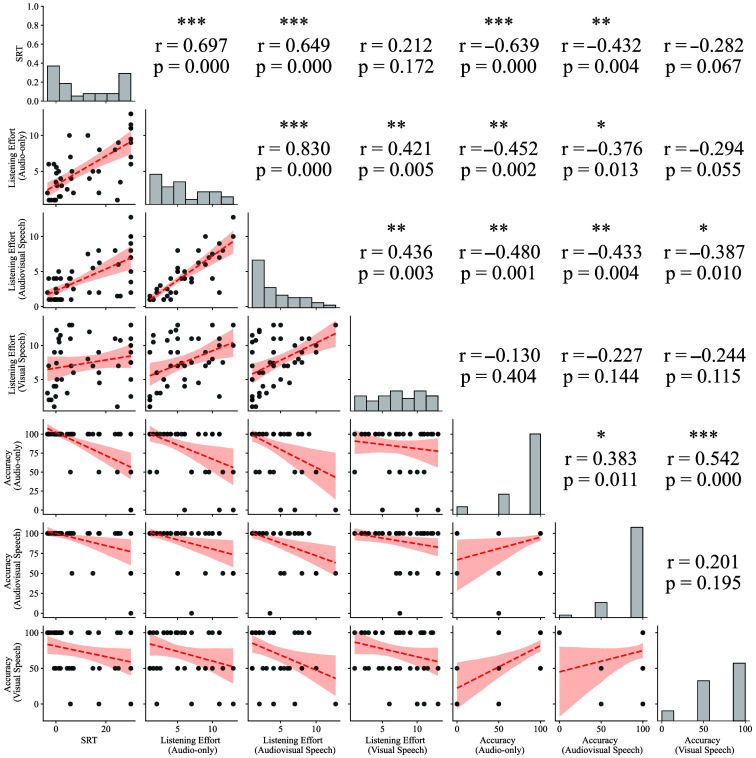
Correlation matrix showing the correlation coefficients between the speech reception threshold (SRT) and behavioral variables (listening effort and accuracy to comprehension questions) in the different listening conditions. Index: ***p≤0.001, **p≤0.01, and *p≤0.05.

We further explored the relationship between behavioral and objective data, as summarized in Fig. 4 in the Appendix. This analysis was an important quality control measure for our study, showing that the objective hearing test results of participants (SRT, adaptive OLSA), their reported listening effort, and their actual performance (task accuracy) were closely related. In other words, participants with higher SRTs (indicating poorer hearing) tended to report higher listening effort and perform the task with lower accuracy.

### 3.2 fNIRS Data

The grand average evoked responses for each group, ROI, and condition are shown in Fig. 3. The corresponding LME analysis revealed differences between the CI patient groups and the control group (Table 3). The follow-up analysis further highlighted differences within the CI groups (Table 4).

**Table 3:** Summary table for the linear mixed-effects (LME) model analysis between NH and CI groups.

	Precentral	Temporal	Occipital
Intercept	−0.58 (−1.22, 0.07)^0.080^	1.13 (−0.15, 2.41)^0.083^	0.11 (−0.12, 0.35)^0.350^
Group (GP)	0.59 (−0.2, 1.39)^0.145^	−0.8 (−2.33, 0.74)^0.307^	−0.2 (−0.49, 0.1)^0.190^
Group (PP)	0.29 (−0.42, 1.0)^0.429^	−0.53 (−1.89, 0.83)^0.446^	−0.1 (−0.36, 0.17)^0.489^
Condition (A)	0.04 (−0.37, 0.44)^0.860^	0.05 (−0.6, 0.71)^0.871^	−0.06 (−0.24, 0.12)^0.522^
Condition (AV)	−0.02 (−0.42, 0.38)^0.926^	0.04 (−0.61, 0.7)^0.896^	0.47 (0.29, 0.66)^**0.000**^
Condition (V)	0.08 (−0.32, 0.49)^0.690^	0.04 (−0.62, 0.69)^0.916^	0.25 (0.06, 0.43)^**0.009**^
Group (GP): condition (A)	0.14 (−0.42, 0.71)^0.619^	0.39 (−0.54, 1.32)^0.408^	0.01 (−0.25, 0.27)^0.945^
Group (PP): condition (A)	0.67 (0.08, 1.26)^**0.025**^	0.28 (−0.67, 1.24)^0.557^	0.07 (−0.2, 0.33)^0.631^
Group (GP): condition (AV)	0.02 (−0.54, 0.59)^0.940^	1.28 (0.36, 2.21)^**0.007**^	−0.29 (−0.55, −0.04)^**0.026**^
Group (PP): condition (AV)	0.86 (0.27, 1.44)^**0.004**^	0.36 (−0.59, 1.31)^0.455^	−0.42 (−0.69, −0.15)^**0.002**^
Group (GP): condition (V)	0.08 (−0.48, 0.65)^0.767^	0.53 (−0.4, 1.45)^0.267^	−0.28 (−0.54, −0.02)^**0.036**^
Group (PP): condition (V)	0.65 (0.06, 1.24)^**0.029**^	0.34 (−0.61, 1.3)^0.477^	−0.28 (−0.55, −0.01)^**0.041**^
Age	0.01 (−0.0, 0.02)^0.106^	−0.03 (−0.05, −0.0)^**0.025**^	−0.0 (−0.01, –0.0)^**0.033**^
Sex	0.16 (−0.14, 0.46)^0.287^	−0.16 (−0.76, 0.44)^0.601^	0.08 (−0.02, 0.18)^0.113^
Etiology	−0.36 (−0.8, 0.09)^0.114^	0.77 (−0.12, 1.65)^0.088^	0.11 (−0.04, 0.27)^0.147^

Model notation: theta (HbO) ∼ group*condition + age + sex + etiology + (1|subject)

Model intercept: group (NH) and condition (rest)

Conditions: audio-only (A), audiovisual speech (AV), and visual speech (V)

Index: “Coefficient (95% CI)^*p* value^”

**Table 4:** Summary table for the linear mixed-effects (LME) model analysis among CI groups.

	Precentral	Temporal
Intercept	−0.26 (−1.4, 0.88)^0.655^	0.56 (−1.8, 2.92)^0.641^
Group (PP)	−0.32 (−0.93, 0.3)^0.312^	0.31 (−0.86, 1.48)^0.602^
Condition (A)	0.18 (−0.29, 0.65)^0.457^	0.45 (−0.36, 1.26)^0.280^
Condition (AV)	0.0 (−0.47, 0.48)^0.992^	1.33 (0.52, 2.14)^**0.001**^
Condition (V)	0.17 (−0.31, 0.64)^0.488^	0.56 (−0.25, 1.37)^0.174^
Group (PP): condition (A)	0.53 (−0.17, 1.22)^0.138^	−0.11 (−1.28, 1.07)^0.858^
Group (PP): condition (AV)	0.83 (0.14, 1.53)^**0.019**^	−0.92 (−2.1, 0.25)^0.124^
Group (PP): condition (V)	0.57 (−0.13, 1.26)^0.111^	−0.18 (−1.36, 0.99)^0.762^
Age	0.01 (−0.01, 0.02)^0.221^	−0.03 (−0.06, 0.0)^0.062^
Sex	0.32 (−0.12, 0.76)^0.158^	−0.27 (−1.19, 0.66)^0.574^
Etiology	−0.3 (−0.86, 0.25)^0.279^	0.78 (−0.35, 1.91)^0.176^

Model notation: theta (HbO)∼group*condition + age + sex + etiology + (1|subject)

Model intercept: group (GP) and condition (rest)

Conditions: audio-only (A), audiovisual speech (AV), visual speech (V)

Index: “Coefficient (95% CI)^*p* value^”

In the NH group, stimulus-specific activation patterns were observed: audio-only stimuli activated the temporal lobe, visual speech stimuli primarily engaged the occipital lobe, and audiovisual stimuli activated both occipito-temporal regions.

In the GP group, the stimulus-specific activation patterns showed alterations compared with NH. Although audio-only stimuli elicited comparable activation in the temporal lobe, visual speech stimuli had significantly weaker GLM coefficients in the occipital ROI (Table 3, p=0.036). In addition, audiovisual stimuli led to significantly stronger activation in the temporal ROI (p=0.007) and significantly weaker activation in the occipital ROI (p=0.026) compared with NH.

In the PP group, stimulus-specific activation patterns differed further: audio-only stimuli induced comparable temporal activation to both NH and GP. Visual speech resulted in significantly weaker activation in the occipital ROI compared to NH (p=0.041), similar to the GP group. For audiovisual stimuli, temporal activation was comparable to NH, but occipital activation was significantly weaker (p=0.002) than NH. Notably, in the precentral ROI, all conditions elicited significantly stronger activation compared with NH, with audiovisual stimuli also showing significantly stronger activation than GP (Table 4, p=0.019).

**Fig. 5:**
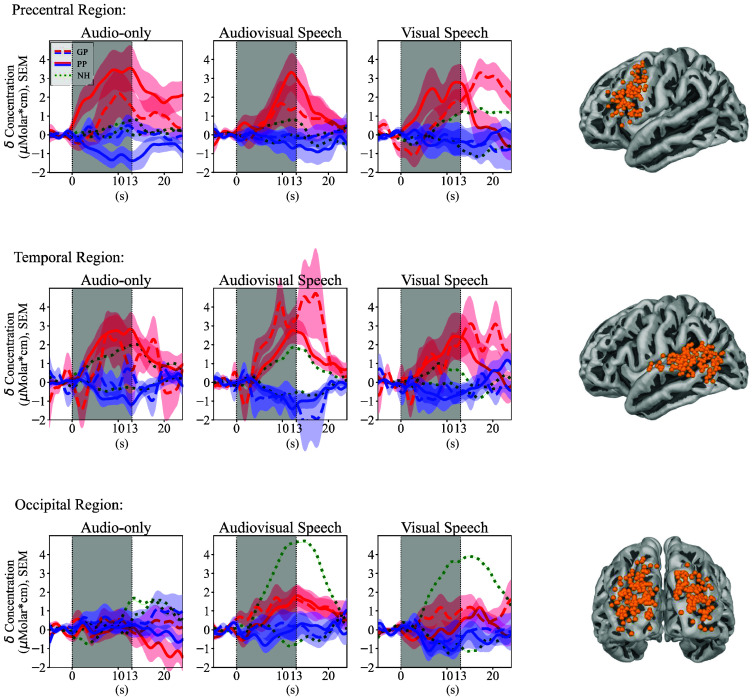
Grand average evoked responses in each region of interest (ROI) for each condition. The red signal represents the concentration change in oxygenated hemoglobin (HbO) and the blue signal in deoxygenated hemoglobin (HbR). Error bars represent the standard error of the mean (SEM). The dashed signals are measured in the good performing (GP), the solid signals in the poor performing (PP), and the green dotted signals in the normal hearing (NH) cohort, who were used as a control in the study. On the right side, we show all registered channel positions that were included in the corresponding ROI, indicating the brain regions contributing to the grand average patterns. We excluded left-handed participants from this plot.

In terms of additional covariates, age was a significant factor in both the temporal and occipital ROIs in the control-group comparison (Table 3), but these were not significant between the CI groups (Table 4). A repeated analysis excluding left-handed participants, detailed in the Appendix (Fig. 5, Tables 6 and 7 in the Appendix), revealed comparable results.

## 4 Discussion

In this study, we used fNIRS to measure brain activation patterns in good or poor performing CI users, as well as NH individuals, during a multimodal speech comprehension task. By adapting the clinically well-established German Matrix Sentence Test (OLSA) across three speech modalities, we were able to identify clear differences. This approach allows for a direct link between brain activation patterns and clinical outcomes, offering a standardized framework for studying speech comprehension in hearing-impaired populations.

### 4.1 Objective and Behavioral Data

Objective and behavioral data are essential to characterize participant performance and to support the interpretation of brain activity.

In the hearing tests, the NH participants achieved perfect WRS and SRTs consistent with normative data for normal hearing individuals.^69^ During the fNIRS task, in the audio-only and audiovisual conditions, they had no difficulty understanding the sentences and reported marginal listening effort. By contrast, during visual speech, they were practically unable to understand the sentences (task accuracy at chance level), which was also shown in the high listening effort ratings and low self-reported lipreading experience.

The GP participants achieved an average SRT score of 1.3 dB SNR and close to 90% WRS. The audio-only and audiovisual conditions were also relatively simple for this group, which was also reflected in the behavioral parameters. Despite their increased listening effort, their accuracy and reaction times to comprehension questions were comparable to NH participants. This suggests that the increased listening effort might be due to the spectrally limited input from the CI,^70^ rather than an inability to understand the sentences. GP participants reported more experience with lipreading than NH participants and demonstrated notable benefits during both audiovisual and visual speech conditions. They had the least listening effort and answered the questions quickest and most accurately of all groups in the visual speech tasks. Further, in audiovisual conditions, many CI users rely on complementary visual cues, such as lip movements, to improve their speech understanding, particularly in challenging listening environments—a phenomenon known as audiovisual gain.^71^ This was observed in our study, with GP participants reporting reduced listening effort in audiovisual conditions compared with audio-only conditions.

For many PP participants, SRTs could not be determined, and their WRS averaged 35%. Behavioral data indicated that this group faced challenges across all conditions. In the audio-only condition, they reported high listening effort and achieved lower task accuracy, although their often short reaction times suggested frequent guessing. The PP participants reported the highest levels of lipreading experience among all groups, with some relying on lipreading as their primary mode of communication. Despite their self-reported experience, PP participants showed marginally lower task accuracy, longer reaction times, and reported higher listening effort, although these differences did not reach statistical significance in the analysis. Nonetheless, these findings suggest that while PP participants are experienced in lipreading, they may still encounter challenges in fully leveraging visual cues for speech understanding. In audiovisual conditions, PP participants demonstrated the strongest audiovisual gain among all groups, as evidenced by lower listening effort ratings and slightly improved task accuracy and reaction times compared with other conditions. This suggests that the integration of auditory and visual cues is particularly beneficial for this group.

### 4.2 fNIRS Data

The analysis of fNIRS data provides insights into the neural mechanisms underlying speech processing and the adaptations in brain activation patterns across participant groups in response to audio-only, visual speech, and audiovisual speech stimuli.

The NH group served as a control in this study, demonstrating the expected stimulus-specific brain activation patterns. Specifically, audio-only stimulation activated the temporal lobe, visual speech stimuli primarily engaged the occipital lobe, and audiovisual stimuli resulted in activation of both regions.

The GP group exhibited brain activity patterns that were often comparable to those of the NH group but also showed notable differences. During audio-only conditions, brain activity in the temporal ROI was similar to that observed in NH participants, indicative of successful hearing rehabilitation. However, during visual speech, we observed significantly weaker activation in the occipital regions, paired with stronger activation in language-related areas such as the temporal and precentral ROIs. In the temporal and precentral ROIs, there was a visually evident activation, with a delayed and prolonged response (see Fig. 3). However, this was not significantly different than the NH data in the statistical analysis (Table 3). The reason for this may be that the delayed pattern was poorly characterized by the hemodynamic response function in the GLM analysis, which expects an earlier peak.^54^ Overall, these findings suggest that expertise in lipreading leads to changes in brain activation patterns. In the case of skillful readers, the process of reading with understanding has been automated. In neural terms, they have an increased efficiency of synaptic connections to carry out the task.^45^ In the case of the GP participants, they may have a similar mechanism, resulting in a more efficient allocation of cortical resources to process speech-related visual information. Previous studies support these findings and suggest an adaptation to visual cues in the occipital cortex of CI users. Using a circular checkerboard pattern in an electroencephalography (EEG) and fNIRS experiment, Chen et al.^22^ found lower visual cortex activation in CI users compared with controls. Using EEG-only, Intartaglia et al.^72^ found a greater contribution of the visual cortex during visual motion changes. Further, the angular gyrus likely plays an important role as a cross-modal hub, integrating and distributing multisensory information between occipital and temporal regions.^73^^,^^74^ As participants become more proficient in lipreading, the angular gyrus might become highly specialized in filtering out the linguistic content of visually perceived information.^48^^,^^75^^,^^76^ We characterize this activity as potentially adaptive, as it represents an additional ability in CI patients to process speech-related information more effectively. During audiovisual conditions, the GP group exhibited stronger activation in the temporal ROI compared with audio-only conditions, indicative of multisensory enhancement in the brain networks responsible for auditory processing.^49^ We also observed weaker activation in the occipital ROI compared with NH, a similar mechanism as seen during visual speech; however, it was only close to significance in the statistical analysis. Overall, these findings highlight the GP group’s ability to integrate auditory and visual cues effectively for improved speech comprehension, leveraging audiovisual gain as a compensatory mechanism.^33^^,^^77^^,^^78^

The PP group showed clear differences from both the NH and GP groups; however, we could not fully confirm our hypothesis. Specifically, in the audio-only conditions in the temporal ROI, the activation was comparable to NH and GP. The temporal regions’ possible reorganization and its impact on speech understanding is an area of active research.^12^^,^^12^^,^^21^^,^^26^^,^^32^^,^^79^^,^^80^ A recent review summarizes that the neural activity in the superior and middle temporal gyrus may be weakened due to the spectrally limited transmission of speech through CIs, but the studies are inconclusive.^81^ To better understand these differences, we need to define narrower ROIs for the auditory region (e.g., isolate the primary auditory cortex). As these regions are small and deeply embedded in the cortex, it is inevitable to use high-density optode arrays and spatial registration of optode positions. Notably, it is often worth considering activation levels in relation to neighboring regions. In this context, we observed that activation in the precentral ROI was as strong as in the temporal ROI for the PP group—an observation not seen in the NH or GP groups. There is an ongoing debate about the exact role of the precentral regions.^82^ In the context of language, this region is primarily responsible for speech repetition and auditory-to-motor mapping.^45^ Further, some studies suggest that they play a crucial role in comprehending speech, but mainly when the sound input is not clear.^47^^,^^82^ In addition, we observed an increase in listening effort during audio-only condition, possibly linked to heightened task-related attention and engagement of the frontally located attention network.^70^^,^^83^ In PP participants, the challenge of understanding speech may lead to broader recruitment of adjacent brain regions.^25^ Overall, the increased involvement of the precentral ROI, along with comparable activation in the temporal ROI, suggests shifts in the allocation of neural resources for adequate speech comprehension. In visual speech conditions, the PP group displayed significantly weaker activation in the occipital ROI, paired with a significantly stronger activation of the precentral and temporal ROIs compared with NH participants. The analysis did not reveal any significant differences between the GP and PP groups. These patterns indicate that PP participants might rely on similar adaptive mechanisms as the GP group to process speech-related visual information effectively. During audiovisual conditions, the PP group exhibited significantly stronger activation in the precentral ROI compared with the NH participants, paired with a significantly weaker activation of the occipital ROI and a comparable activation of the temporal ROI. The patterns observed in the precentral and occipital ROIs during audiovisual tasks were similar to those seen during visual speech, suggesting that PP participants rely heavily on visual processing even in the presence of auditory input. This reliance underscores the importance of audiovisual gain as a compensatory mechanism, although it is more heavily weighted toward visual processing in this group compared to the GP group.

### 4.3 Developmental Aspects

In clinical practice, it is well established that earlier implantation leads to better outcomes.^15^^,^^84^

This is primarily due to the existence of a sensitive period for language acquisition in children, which is linked to critical neurodevelopmental processes such as synaptogenesis and the myelination of long fiber tracts. Implantation outside this sensitive period can result in delayed maturation of the auditory cortex^15^^,^^84^^,^^85^ and a lack of critical early exposure to language.^86^^–^^88^

Our study population included both prelingually and postlingually deafened CI users. We controlled for this in the statistical analysis and found no statistically significant effect of this variable. However, it is important to note that while our analysis did not identify significant effects, this does not contradict the well-documented neurophysiological differences reported in the literature. 

Most previous studies on the developmental aspects of CI relied on electrophysiology. By contrast, fNIRS measures cortical hemodynamic activity (rather than electrical potentials); therefore, it indirectly reflects local neural activity.^43^ As such, the extent to which fNIRS can capture the neurodevelopmental differences between prelingually and postlingually deaf CI users remains an active area of research.^89^

### 4.4 Limitations

One limitation of our study is that the control group we used does not match the test group in terms of age and sex, which could affect the brain activity we measured.^90^ To address this limitation, we included age and sex as additional covariates to the LME model. This analysis showed no significant effects of sex on brain activity; however, in one of the analyses (Table 3), in the temporal and occipital ROI, age was a significant contributor (p=0.025 and p=0.033). This was inconsistent among other brain regions and was not significant in the follow-up analysis (Table 4). The possible influence may be explained by age and sex specific factors such as tissue optical properties, affecting the light penetration of near-infrared light.^91^

Another challenge in fNIRS studies is the variation in the positions of the optodes.^92^ To address this issue, we used a 3D scanner to register the optode positions and mapped them onto the AAL atlas. However, it would be more accurate to map the channels to individual anatomies. For a considerable part of the study population, however, anatomical information on the brain from magnetic resonance imaging (MRI) was not available. Further, to define smaller ROIs more precisely in future studies, we recommend using a high-density optode array and performing the reconstruction of brain activity on individual MRIs. This approach can lead to increased spatial resolution and, consequently, more reliable single-subject results in fNIRS studies.^44^^,^^93^^–^^95^

## 5 Conclusion

This study examined brain activity in CI users with different speech comprehension abilities during a clinically adapted audiovisual speech comprehension task, comparing their performance to NH controls and supporting findings with hearing tests, questionnaires, and comprehension assessments. NH participants excelled in audio-only and audiovisual tasks but struggled with visual speech. GP participants showed balanced performance across conditions, benefiting from their lipreading experience, whereas PP participants faced difficulties in all tasks and relied heavily on visual speech cues. Neural activation patterns reflected these differences: NH participants exhibited the expected stimulus-specific activation. GP participants showed comparable temporal activation to NH participants in audio-only conditions, which is indicative of successful hearing rehabilitation. During visual speech processing, weaker activation was observed in the occipital region, but stronger activation in language-related regions, suggesting effective processing of visual speech cues. They also demonstrated effective multisensory integration in audiovisual tasks. PP participants exhibited altered neural resource allocation in audio-only condition, suggesting shifts toward the precentral regions that were not observed in GP or NH participants. During visual speech, they demonstrated a comparable processing of visual speech cues to GP. However, in audiovisual conditions, they showed a more pronounced dependence on visual speech cues than GP.

By continuing to improve our understanding of the neural mechanisms in CI users with different levels of speech understanding, we will be able to provide better preoperative planning and postoperative rehabilitation to improve CI outcomes.

## 6 Appendix

Table 5 shows detailed information on participants demographics, summarized in Table 2 on a group level.

**Table 5:** Participant demographics.

ID	Sex	Age	SQ	Hearing tests		Medical history					Questionnaires	
				WRS (%)	SRT	Cause of hearing loss	Age at deafness	Age at (first) implantation	Etiology	CI side	Handedness	Lipreading
S01	F	73	11.6	90	6.4	Meniere	43	63	Post	R	1	1
S02	F	64	7.2	95	1.8	Unknown	19	54	Post	R	−0.5	6
S03	F	51	10.0	95	0.2	Unknown	11	48	Post	R	1	2
S04	F	60	9.7	60	5.4	Unknown	20	57	Post	R	1	3.5
S05	F	68	2.4	95	−0.7	Unknown	52	61	Post	L	1	1.5
S06	F	66	16.6	90	5.8	Unknown	26	59	Post	L	1	4
S07	F	52	5.6	90	−0.8	Unknown	16	36	Post	R	1	8
S08	F	72	10.4	75	4.5	Otosclerosis	38	52	Post	L	1	6
S09	M	63	14.6	80	0.2	Meningitis	43	53	Post	L	1	2
S10	F	67	8.0	90	0.4	Unknown	33	60	Post	L	1	6
S11	F	52	7.1	80	6.1	Unknown	4	39	Post	R	–1	8
S12	F	53	4.9	95	2.3	Meningitis	4	41	Post	R	1	10
S13	F	57	29.6	55	12.8	Meningitis	3	37	Pre	R	1	9
S14^a^	F	75	13.2	70	1.2	Unknown	54	74	Post	R	0	8
S15	M	26	3.5	100	−3.4	Congenital	<1	5	Pre	R	1	7
S16	M	72	27.0	85	13.4	Trauma	63	63	Post	R	1	2
S17	M	29	2.9	95	−1.9	Congenital	1	3	Pre	R	1	6
S18	M	33	2.6	75	1.1	Congenital	<1	2	Pre	R	1	6
S19	F	54	8.2	50	17.4	Congenital	4	48	Post	L	1	8.5
S20	F	67	8.3	90	1.9	Unknown	<1	47	Pre	L	1	5
S21	M	70	24.6	95	−0.6	Unknown	20	53	Post	L	1	2
S22	F	28	2.2	95	−0.1	Unknown	5	10	Post	R	1	7
S23	M	66	16.2	0	max	Congenital	1	47	Pre	R	1	8
S24	F	36	3.2	100	1.3	Congenital, otitis media	4	27	Post	L	1	3
S25	F	26	2.3	85	0	Congenital	6	17	Post	L	1	6
S26	F	26	2.8	100	−2.8	Congenital	2	3	Pre	L	1	8
S27	F	60	14.3	45	17.1	Otosclerosis	20	55	Post	L	1	5
S28	M	78	21.4	80	7	Otitis media	68	77	Post	R	1	0
S29	M	25	4.6	55	24.6	Congenital	<1	11	Pre	L	−0.4	8
S30	M	22	3.5	100	−2.9	Infection during pregnancy	<1	1	Pre	R	−1	7
S31	M	69	11.2	20	17.2	Congenital	5	50	Post	L	1	8
S32	F	56	8.0	45	23.7	Trauma	25	50	Post	L	1	7
S33^a^	M	67	15.8	85	0.9	Unknown	55	55	Post	R	1	0
S34	M	42	4.5	0	max	Congenital	<1	38	Pre	L	1	9
S35	F	33	4.3	10	max	Congenital	<1	11	Pre	R	0.6	8
S36	F	71	12.4	45	max	Trauma	25	47	Post	L	1	9
S37	M	68	19.1	50	14.8	Asphyxia at birth	<1	61	Pre	R	1	8
S38^a^	F	19	1.4	45	max	Congenital	<1	1	Pre	L	1	9
S39	M	70	5.3	25	max	Acoustic neuroma	64	64	Post	R	1	3
S40	M	71	7.7	50	max	Meningitis	55	55	Post	R	1	3
S41	M	53	4.2	20	max	Unknown	<1	23	Pre	R	1	7
S42	F	46	4.4	0	max	Unknown	<1	39	Pre	L	–1	10
S43	M	69	16.8	45	25	Meningitis	1	56	Pre	L	1	9.5
S44	F	33	3.3	25	25.8	Meningitis	<1	3	Pre	R	1	9.5
S45	F	70	3.9	30	max	Meningitis	62	62	Post	L	1	7
S46	F	39	2.8	30	max	Unknown	<1	12	Pre	R	0.7	8

SQ: signal quality based on cardiac component.

WRS: results of the Freiburg monosyllabic word test at 65 dB sound pressure level (SPL).

SRT: speech reception threshold (SRT) using the adaptive OLSA test.

Age at deafness: self-reported age at which the hearing loss could not be compensated with hearing aids.

Age at implantation: the age at which the participant received the implant (in bilateral cases, the first implant).

Etiology: whether the hearing loss occurred before or after learning language (defined at a maximal age of 4).

CI Side: the side used during fNIRS measurement.

Handedness: a value close to 1 means right-handed, close to 0 ambidextrous, and close to (–1) left-handed.

Lipreading: self-reported lipreading experience on a scale from 0 to 10.

a Excluded participants.

Figure 4 shows the correlation between the behavioral and objective data in our study and serves as a quality control measure. Figure 5 shows the grand average evoked responses in each ROI for each condition. It is homologous to Fig. 3, except that the left-handed participants have been excluded to confirm that the results remain comparable regardless of handedness.

Tables 6 and 7 represent a repeated linear mixed-effects (LME) model analysis, and they’re analogous to Tables 3 and 4, except that the left-handed participants have been excluded to confirm that the results remain comparable regardless of handedness. Table 8 is a supplementary analysis of the behavioral variables, supporting the findings from Fig. 2 with T-statistics.

**Table 6:** Summary table for the linear mixed-effects (LME) model analysis between NH and CI groups, excluding left-handed participants.

	Precentral	Temporal
Intercept	−0.59 (−1.26, 0.08)^0.086^	1.1 (−0.23, 2.43)^0.106^
Group (GP)	0.64 (−0.21, 1.49)^0.140^	−0.69 (−2.33, 0.95)^0.410^
Group (PP)	0.28 (−0.49, 1.04)^0.477^	−0.47 (−1.93, 0.99)^0.528^
Condition (A)	0.01 (−0.41, 0.44)^0.951^	0.06 (−0.63, 0.74)^0.873^
Condition (AV)	0.02 (−0.41, 0.44)^0.937^	0.05 (−0.64, 0.74)^0.886^
Condition (V)	0.07 (−0.36, 0.49)^0.749^	0.04 (−0.65, 0.72)^0.915^
Group (GP): condition (A)	0.18 (−0.42, 0.78)^0.551^	0.4 (−0.59, 1.38)^0.427^
Group (PP): condition (A)	0.73 (0.11, 1.34)^**0.020**^	0.32 (−0.68, 1.32)^0.534^
Group (GP): condition (AV)	−0.03 (−0.63, 0.57)^0.924^	1.28 (0.29, 2.26)^**0.011**^
Group (PP): condition (AV)	0.84 (0.22, 1.46)^**0.007**^	0.31 (−0.69, 1.31)^0.546^
Group (GP): condition (V)	0.09 (−0.51, 0.69)^0.768^	0.52 (−0.46, 1.51)^0.300^
Group (PP): condition (V)	0.66 (0.05, 1.28)^**0.035**^	0.28 (−0.72, 1.27)^0.588^
Age	0.01 (−0.0, 0.02)^0.169^	−0.03 (−0.05, −0.0)^**0.020**^
Sex	0.19 (−0.12, 0.51)^0.231^	−0.1 (−0.74, 0.54)^0.753^
Etiology	−0.35 (−0.82, 0.11)^0.140^	0.79 (−0.14, 1.71)^0.096^

Model notation: theta (HbO)∼group*condition + age + sex + etiology + (1|subject)

Model intercept: group (NH) and condition (rest)

Conditions: audio-only (A), audiovisual speech (AV), and visual speech (V)

Index: “Coefficient (95% CI)^*p* value^”

**Table 7:** Summary table for the linear mixed-effects (LME) model analysis between CI groups, excluding left-handed participants.

	Precentral	Temporal
Intercept	−0.26 (−1.48, 0.97)^0.682^	0.58 (−1.96, 3.12)^0.653^
Group (PP)	−0.38 (−1.03, 0.27)^0.251^	0.26 (−0.99, 1.5)^0.688^
Condition (A)	0.2 (−0.32, 0.71)^0.455^	0.46 (−0.42, 1.33)^0.307^
Condition (AV)	−0.01 (−0.53, 0.5)^0.963^	1.33 (0.45, 2.2)^**0.003**^
Condition (V)	0.16 (−0.35, 0.67)^0.543^	0.56 (−0.32, 1.43)^0.211^
Group (PP): condition (A)	0.55 (−0.2, 1.29)^0.151^	−0.08 (−1.34, 1.17)^0.897^
Group (PP): condition (AV)	0.87 (0.12, 1.61)^**0.022**^	−0.97 (−2.22, 0.28)^0.129^
Group (PP): condition (V)	0.57 (−0.17, 1.32)^0.132^	−0.24 (−1.5, 1.01)^0.702^
Age	0.01 (−0.01, 0.02)^0.337^	−0.03 (−0.06, 0.0)^0.056^
Sex	0.38 (−0.1, 0.85)^0.121^	−0.18 (−1.19, 0.84)^0.733^
Etiology	−0.28 (−0.86, 0.3)^0.341^	0.81 (−0.39, 2.0)^0.186^

Model notation: theta (HbO) ∼ group*condition + age + sex + etiology + (1|subject)

Model intercept: group (GP) and condition (rest)

Conditions: audio-only (A), audiovisual speech (AV), and visual speech (V)

Index: “Coefficient (95% CI)^*p* value^”

**Table 8:** T-statistics for behavioral variables.

Variable	Condition	Group 1	Group 2	T-statistic	P value	Corrected P value (Bonferroni)
Listening effort	Audio-only	NH	GP	−4.595	<0.001	<0.01
Listening effort	Audio-only	NH	PP	−8.478	<0.001	<0.001
Listening effort	Audio-only	GP	PP	−4.691	<0.001	<0.01
Listening effort	Audiovisual speech	NH	GP	−4.168	<0.001	<0.01
Listening effort	Audiovisual speech	NH	PP	−6.915	<0.001	<0.001
Listening effort	Audiovisual speech	GP	PP	−4.688	<0.001	<0.01
Listening effort	Visual speech	NH	GP	1.850	0.072	1.0
Listening effort	Visual speech	NH	PP	0.968	0.339	1.0
Listening effort	Visual speech	GP	PP	−0.967	0.34	1.0
Reaction time	Audio-only	NH	GP	−2.680	<0.05	0.337
Reaction time	Audio-only	NH	PP	−4.703	<0.001	<0.01
Reaction time	Audio-only	GP	PP	−1.460	0.152	1.0
Reaction time	Audiovisual speech	NH	GP	−2.646	<0.05	0.316
Reaction time	Audiovisual speech	NH	PP	−4.070	<0.001	<0.05
Reaction time	Audiovisual speech	GP	PP	−2.793	<0.01	0.27
Reaction time	Visual speech	NH	GP	0.105	0.917	1.0
Reaction time	Visual speech	NH	PP	−2.670	<0.05	0.359
Reaction time	Visual speech	GP	PP	−2.534	<0.05	0.447
Accuracy	Audio-only	NH	GP	1.000	0.328	1.0
Accuracy	Audio-only	NH	PP	3.901	<0.001	<0.05
Accuracy	Audio-only	GP	PP	3.522	<0.01	0.053
Accuracy	Audiovisual speech	NH	GP	1.000	0.328	1.0
Accuracy	Audiovisual speech	NH	PP	2.666	<0.05	0.412
Accuracy	Audiovisual speech	GP	PP	2.216	<0.05	0.993
Accuracy	Visual speech	NH	GP	−3.758	<0.001	<0.05
Accuracy	Visual speech	NH	PP	−2.128	<0.05	1.0
Accuracy	Visual speech	GP	PP	1.295	0.204	1.0

## Data Availability

The measurement data would be made available upon request to the authors after a formal data-sharing agreement has been reached.
